# Identification of Single and Combined Serum Metabolites Associated with Food Intake

**DOI:** 10.3390/metabo12100908

**Published:** 2022-09-27

**Authors:** Therese Karlsson, Anna Winkvist, Millie Rådjursöga, Lars Ellegård, Anders Pedersen, Helen M. Lindqvist

**Affiliations:** 1 Department of Internal Medicine and Clinical nutrition, Institute of Medicine, Sahlgrenska Academy, University of Gothenburg, Box 115, 405 30 Gothenburg, Sweden; 2 Department of Biology and Biological Engineering, Division of Food and Nutrition Science, Chalmers University of Technology, 412 96 Gothenburg, Sweden; 3 Department of Public Health and Clinical Medicine, Sustainable Health, Umeå University, 901 87 Umeå, Sweden; 4 Clinical Nutrition Unit, Department of Gastroenterology, Sahlgrenska University Hospital, 43145 Gothenburg, Sweden; 5 Swedish NMR Centre, University of Gothenburg, Box 465, 405 30 Gothenburg, Sweden

**Keywords:** ^1^H-NMR metabolomics, food intake, serum metabolites, 4-day dietary record, habitual food intake

## Abstract

Assessment of dietary intake is challenging. Traditional methods suffer from both random and systematic errors; thus objective measures are important complements in monitoring dietary exposure. The study presented here aims to identify serum metabolites associated with reported food intake and to explore whether combinations of metabolites may improve predictive models. Fasting blood samples and a 4-day weighed food diary were collected from healthy Swedish subjects (*n* = 119) self-defined as having habitual vegan, vegetarian, vegetarian + fish, or omnivore diets. Serum was analyzed for metabolites by ^1^H-nuclear magnetic resonance spectroscopy. Associations between single and combined metabolites and 39 foods and food groups were explored. Area under the curve (AUC) was calculated for prediction models. In total, 24 foods or food groups associated with serum metabolites using the criteria of rho > 0.2, *p* < 0.01 and AUC ≥ 0.7 were identified. For the consumption of soybeans, citrus fruits and marmalade, nuts and almonds, green tea, red meat, poultry, total fish and shellfish, dairy, fermented dairy, cheese, eggs, and beer the final models included two or more metabolites. Our results indicate that a combination of metabolites improve the possibilities to use metabolites to identify several foods included in the current diet. Combined metabolite models should be confirmed in dose–response intervention studies.

## 1. Introduction

Poor diet is one of the leading risk factors for morbidity and mortality worldwide [1,2]. Nutritional epidemiology provides the main method for the assessment of long-term risk from diet in populations and accurate measurements of habitual diet is therefore crucial. However, measurement of true dietary intake is challenging. Traditionally used methods include food frequency questionnaires (FFQ), 24 h recalls and food records (weighed or unweighted). These methods are based on self-reported data and therefore prone to subjectivity and suffer from systematic and random errors such as recall bias and under-reporting [3]. As a result, epidemiological studies of diet/health relationships may suffer from inaccurate risk estimates and/or inconsistencies [3,4]. Consequently, objective measures, for example biomarkers, are of importance to reduce errors in the measure of dietary exposure and to correlate food intake to health outcomes.

Few validated dietary biomarkers exist and these include urinary sodium, nitrogen and sucrose/fructose for the estimation of salt, protein, and sugar intake, respectively, and the doubly labeled water technique for energy expenditure and alkylresorcinols for whole grain intake [4]. Nevertheless, putative dietary biomarkers have been proposed for several foods or food groups, including red meat, coffee, nuts, red wine, vegetables, legumes, fermented food products, citrus fruit, apples, milk, cheese, tea, and sugar-sweetened beverages [5,6,7,8,9,10,11,12,13] as well as for dietary patterns [14]. Metabolomics has been used to identify several of these dietary biomarkers and seems to be a promising approach for providing possible biomarkers of dietary intake [4]. Commonly, single biomarkers have been identified to estimate intake of a food, food groups, or nutrients. However, combining biomarkers may further improve the possibility to accurately predict dietary exposure. Using observational and intervention studies, multiple biomarkers as predictors of dietary exposure have been explored [15]. Even so, the food, food groups, and dietary patterns explored are to date limited.

Using ^1^H-nuclear magnetic resonance (^1^H-NMR) metabolomics analysis, we aimed to (1) identify serum metabolites associated with reported food intake and (2) explore the potential to use combinations of serum metabolites to improve predictive models of reported habitual food intake.

## 2. Results

### 2.1. Subject Characteristics

Subjects ranged in age from 19 to 57 years with a median age of 28 years, 63% of the subjects were female, and the median body mass index (BMI) (min-max) was 21.6 (18.2–28.9) kg/m^2^. The distribution of self-reported habitual dietary intake pattern was vegan (*n* = 43), lacto–ovo vegetarian (*n* = 25), lacto–ovo vegetarian + fish (*n* = 13), and omnivore (*n* = 38). For most food groups, consumers, as well as non-consumers, were identified; in short, 66% of subjects reported consumption of cruciferous vegetables, 26% of fish and shellfish, 31% of meat and poultry, 44% of eggs, 60% of dairy, and 23% reported consumption of beer during the 4 days. Relatively few subjects reported consuming soybeans (3.4%), white wine (14%), or spirits (5.9%).

### 2.2. Diet-Metabolite Associations

In total, 438 associations with absolute rho > 0.2 (rho = −0.506 to 0.628) and *p* < 0.01 were identified between the 39 foods/food groups and 237 ^1^H-NMR -variables reflecting serum metabolites. These are presented in Supplemental Table S1.

Adding the criteria AUC ≥ 0.7, 95 of these associations, which included 24 of the food or food groups, remained of interest (Table 1). Reported consumption (non-consumers and consumers) of these 24 foods/food groups are shown in Supplemental Table S2.

#### 2.2.1. Plant-Derived Foods

No associations with metabolites were identified for legume consumption, but consumption of soy beverages (rho = 0.32, AUC = 71%) and soy products (rho = 0.44, AUC = 78%) was associated with glycine (Table 1). In addition, soybean consumption was associated with asparagine and valine; however, among the study subjects, overall consumption of soybeans was low (3.4%). Diet-metabolite associations were also identified for cruciferous vegetables and citrus fruits. Cruciferous vegetable consumption was associated with glutamine + an unidentified metabolite. Citrus fruits and citrus products were associated with two unidentified variables, and this was also the case for the reported consumption of nuts and almonds (Table 1).

#### 2.2.2. Animal-Derived Foods

Food-metabolite associations for meat, poultry, eggs, and dairy consumption were mainly characterized by amino acids (Table 1). Consumption of red meat, meat products/processed meat, poultry, eggs, and total dairy were predominantly associated with the branched-chain amino acids (BCAAs) valine, leucine, and isoleucine (rho = 0.25–0.44, AUC = 70–77%). These foods also were associated with 3-hydroxybutyrate (rho = 0.37–0.40, AUC = 75–82%) and creatine (rho = 0.27–0.54, AUC = 71–87%). In addition, consumption of red meat was associated with lipids, phosphocholine, acetylcholine, and phosphoethanolamine (rho = 0.27, AUC = 70%) and meat products/processed meat were associated with creatinine (rho = 0.303, AUC = 71%). Cheese and fermented dairy products were associated with 2-aminobutyrate (uncertain identification) but not with 3-hydroxybutyrate. Total fish and seafood consumption were also associated with creatine (rho = 0.41, AUC = 75%), creatine + lysine (rho = 0.44, AUC = 77%), and 3-hydroxybutyrate (rho = 0.30, AUC = 71%).

#### 2.2.3. Alcoholic Beverages

Beer consumption was associated with several metabolites: isoleucine + unidentified; lipids/free fatty acids + methylguanidine; and glucose + lysine + unidentified and proline (rho = 0.24–0.38, AUC = 71–80%).

### 2.3. Combined Serum Metabolites for Prediction of Food Intake

To improve the prediction of reported food exposure by serum metabolites, combinations of two or more metabolites were created. Combinations of metabolites were selected for all 39 food/food groups using forward stepwise logistic regression. For soybeans, citrus fruits and marmalade, nuts and almonds, green tea, red meat, poultry, total fish and shellfish, dairy, fermented dairy, cheese, eggs, and beer the final regression model included two or more metabolites (Table 2).

**Figure 1:**
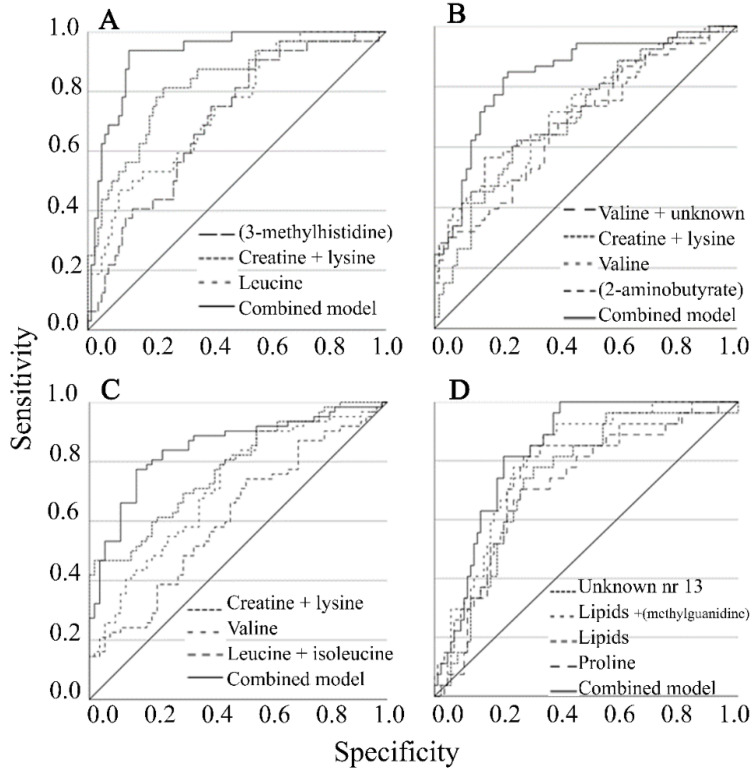
Area under the curve for single metabolites and combined metabolite models for (**A**) red meat, (**B**) eggs, (**C**) fermented dairy, (**D**) beer. Diagonal line represents the reference line.

AUC for soybean consumption was improved using combined metabolites (valine and asparagine). For citrus fruits and marmalade as well as for nuts and almonds, combined metabolites (all unidentified) only marginally improved the model (Table 2); note that unidentified variables may originate from the same metabolite. The predictive ability of consumption of meat products/processed meat and poultry consumption increased when combining metabolites (creatinine, creatine + lysine, and valine). For red meat consumption, the combined metabolite model (3-methylhistidine, leucine, and creatine + lysine) improved the AUC (92%) compared to each separate metabolite (70–82%) (Figure 1A).

For egg consumption, the combined metabolite model improved AUC (84%) in comparison to models with each separate metabolite (AUC = 68–72%) (Table 2, Figure 1B). For fermented dairy products, the combined metabolite model improved AUC (85%) in comparison to models with each separate metabolite (AUC = 61–77%) (Table 2, Figure 1C). Finally, for beer consumption, the combined metabolite model improved AUC (84%) in comparison to models with each separate metabolite (AUC = 71–80%) (Table 2, Figure 1D).

## 3. Discussion

The results from our explorative analysis show that serum metabolites can be associated to reported intake of different foods, when applying partial correlation and stepwise forward logistic regression analysis. In addition, the use of a combination of several serum metabolites improved predictive models for several foods and food groups, including soybeans, meat products/processed meat, poultry, red meat, eggs, fermented dairy, and beer.

Comparing the predictive power of our combined metabolite models with the prediction by one metabolite (AUC) from a paper by Wang et al. [16] shows that the predictability was higher in our combined metabolite models for soy beans, red meat, poultry, total meat, eggs, and beer.

Combining metabolites using stepwise logistic regression analyses have only been used in a few previous studies. In contrast to our study, these studies aimed at exploring associations between intake of specific foods (cocoa [17], bread [18], red wine [11], and walnuts [19]) and changes in urine metabolites. In addition, the dietary assessment methods used in these studies were FFQs to capture habitual intake, while a 4-day dietary record to capture recent intake was used in the current analyses. Even though both methods may capture habitual intake, dietary records also reflect the specific foods actually consumed the days before sampling, and this is an advantage when aiming to identify patterns of metabolites in serum associated with food consumption.

We did not identify some of the known food-metabolite associations such as, for example, genistein and daidzein for soy consumption [7]. This reflects that ^1^H-NMR metabolomics, the method used for our serum analysis, is a less sensitive method than for example mass spectrometry and therefore hampered the identification of low concentration metabolites. 

Soy products are becoming more common in our diets due to the protein shift from animal-based products to plant-based products. In this study sample, with a large proportion of vegans and vegetarians, about 56% reported consuming soy products, and these reported intakes were associated with a high content of the amino acid glycine in serum. Glycine is a nonessential amino acid which can be found in many types of foods [20]. However, soy protein has a glycine content of 3.8 g/100 g, which is about twice the content of other foods also regarded to have a high glycine content [21]. Although many subjects consumed soy products, few subjects consumed soybeans. Surprisingly, in our study, soybeans did not associate with glycine but with valine and asparagine. However, the content of glycine in dried and cooked soybeans is only about 0.46 g/100 g, which probably explains our result. This highlights the complexity of using food groups when trying to identify markers for food intake. In our study, a combination of valine and asparagine improved the specificity to capture the intake of soybeans. Legumes and nuts, including soybeans, have a comparatively high content of valine and asparagine, but foods from animal sources also contribute to these amino acids, thus complicating the picture. 

In a combined metabolite model to predict intake of green/herbal tea, glycine and asparagine jointly qualified. It is unlikely that these amino acids would increase after green/herbal tea intake, and we therefore tested if subjects who consumed soy products also were more prone to drink green/herbal tea. This was not the case, but intake of green/herbal tea may be correlated to consumption of some other foods that we were unable to capture or this could be a chance finding due to multiple testing.

Reported intake of cruciferous vegetables was associated with a variable including glutamine and an unidentified metabolite. This may be explained by the finding that subjects with a high intake of cruciferous vegetables concurrently had a low intake of protein. Similarly, serum glutamine has been reported to be inversely correlated to protein intake in other studies [22]. In line with this, glutamine has been associated with vegan diet in a previous report [23]. A higher glutamine-to-glutamate ratio has been associated with an improved cardiometabolic risk profile, making this marker interesting to explore further [24]. Nevertheless, a direct link between dietary intake pattern and serum glutamine/glutamate ratio has not yet been established.

In our study, foods from animal sources were associated with a wide range of amino acids, most notably the branched amino acids leucine, valine, and isoleucine and creatine. Interestingly, most animal protein sources were associated with 3-hydroxyisobutyrate, although this metabolite did not qualify in any of the combined metabolite models.

When comparing models among foods of different animal sources, creatinine was only included in the model for processed meat and 3-methylhistidine was only included in the model for red meat. Still, both metabolites were associated with not only meat intake but also with muscle mass in our data. When adjusting for lean body mass in the analyses, none of these markers remained significant, suggesting endogenous origin, and hence cannot be seen as reliable markers for meat intake. In contrast to our findings, 3-methylhistidine has previously been associated with poultry intake [25,26]. The difference in results may be explained by the adjustment for lean body mass in our study. To improve models further, the combination of serum metabolites with lipidomic data or with fatty acid concentrations may be applied since many amino acids are overlapping among animal products.

In contrary to other animal-foods, the models for eggs and cheese contained the metabolite 2-aminobutyrate (L-alpha-aminobutyric acid), which is a metabolite from the amino acid metabolism. Few studies have reported 2-aminobutyrate concentrations in relation to diet. However, Gu et al. found increased concentrations of 2-aminobutyrate in relation to a diet high in eggs and other animal proteins [27].

Beer consumption was associated with several metabolites, among them lipids, glucose, and proline. In a combined metabolite model, lipids/free fatty acids, proline, and a variable including isoleucine + unidentified increased the precision. Since accurate reported intake of alcoholic beverages is notoriously difficult to obtain, this could be of special interest for future research. In our setting, beer consumption 1–3 days before sampling had a significant impact on blood lipids, a fact that might be of importance when studying blood lipids and for clinical sampling. In support of our results, previous studies have shown an association between alcohol intake and increased levels of high-density lipoprotein levels [28,29]. In our study, for other alcoholic beverages only single metabolites were associated, presumably xylose, for white wine and citrate and a combined variable with lactate, proline and 3-hydroxybuturate for spirits. Contrary to our results, in a meta-analysis of three cohorts the reported alcohol intake was inversely correlated with citrate in serum samples [30]. However, in our study alcohol could be consumed up to the day before sampling, whereas the number of days between consumption of alcohol and sample collection was unknown in the study by Würtz et al. [30].

Since metabolites from certain foods have different half-times in serum, a combination of several metabolites may jointly cover both short- and long-term metabolites, resulting in a more accurate picture of the dietary intake. In addition, the effect of endogenous metabolites may have less influence on the total concentration using a combination of several markers. 

This study has several strengths. Fasting serum samples were used and these were rigorously handled following a strict protocol, resulting in high-quality ^1^H-NMR data. The wide range of consumption of food specifically from animal sources is another strength, as it makes it possible to find correlations between concentration of metabolites and specifically meat but also dairy and egg. In a normal population, few individuals exclude meat. However, this is also a weakness of the study as there can be other lifestyle factors coincident among vegans in addition to excluding meat, fish, egg, and dairy that might influence the results. Therefore, findings from this study should be confirmed in other settings.

To sum up, using models with combinations of metabolites quantified by ^1^H-NMR metabolomics analysis shows the potential to improve the precision of assessing certain food intake compared to today’s standard subjective methods, such as FFQ. It is important to notice that when a certain food represents a minor portion of all the carbohydrates, proteins, lipids, or overall calories, it is unlikely its individual fingerprint can be identified by ^1^H-NMR metabolomics analysis in serum since metabolites unique for individual foods are often found in low concentrations. 

Findings in this work should be confirmed in intervention studies and evaluated in large epidemiological cohorts.

## 4. Materials and Methods

### 4.1. Subjects

The current work was based on data from a cross-sectional metabolomics study which included 124 healthy subjects living in the Gothenburg area, Sweden, registered at Clinicaltrials.gov as NCT02039609. Recruitment, study design, subject characteristics, and dietary intake have been described in detail elsewhere [31,32]. Briefly, healthy females and males complying with self-reported habitual vegan, (lacto-/ovo-) vegetarian, vegetarian adding fish, or omnivore diets were recruited during 2013 and 2015. Inclusion criteria were age between 18 and 65 y, no regular use of medications, and having a BMI between 18 and 30 kg/m^2^. Subjects who were pregnant, lactating, or used nicotine products regularly were excluded. Subjects were not allowed to drink alcohol the night before or to consume diet supplements one week before sampling.

**Figure 2:**
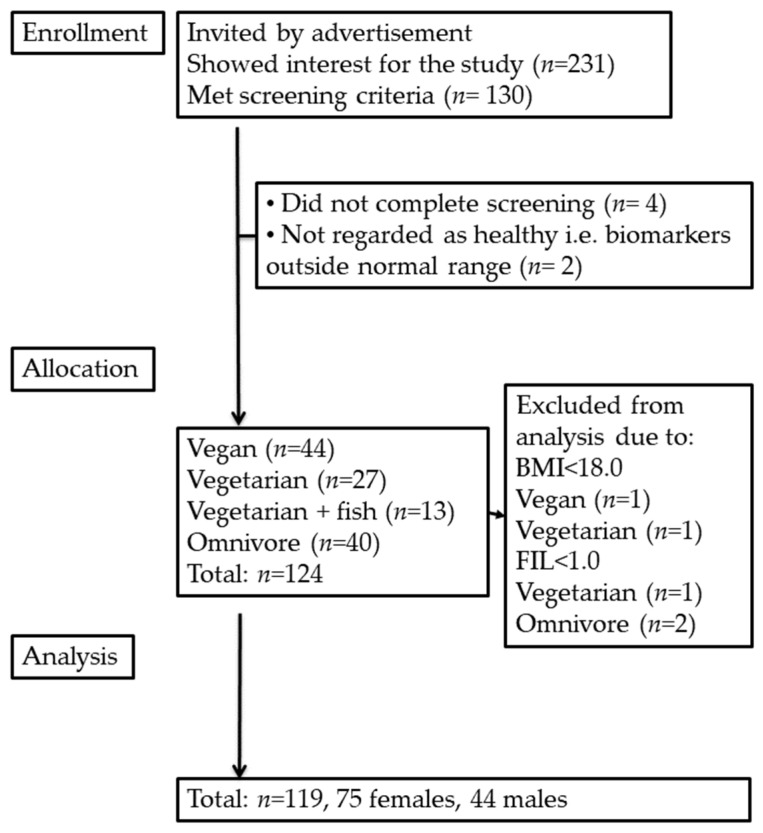
Study flowchart. Body mass index, BMI; food intake level, FIL.

Subjects with BMI < 18.0 kg/m^2^ (*n* = 2) or with food intake level (FIL; calculated by dividing total reported energy intake with estimated basal metabolic rate [33]) < 1.0 (*n* = 3) were excluded, leaving 119 subjects for the current analyses (Figure 2).

### 4.2. Dietary Assessment

Food intake was estimated from a 4-day weighed food diary recorded by subjects at one time point before blood sampling. Subjects were instructed to weigh all food and drinks consumed during 3 weekdays and 1 weekend day using a household scale. Records were registered in the nutritional calculation program DietistNet version 18.12.16, (Kost och Näringsdata AB, Bromma, Sweden). For nutritional calculations the databases from Sweden (National Food Agency, Uppsala, Sweden, version 17.12.15) and Finland (Fineli, National Institute for Health and Welfare, Helsinki, Finland, version 18.02.28) were used. Micro- and macro-nutrient intakes have been reported in detail elsewhere [32].

Dietary intake was categorized into 32 foods/food groups and 7 combined groups such as total meat (Supplemental Table S1). The foods/food groups have all previously been associated with different metabolites in serum in metabolomic studies [6,7,8,9,10,26,34,35].

### 4.3. Covariate Data

Weight and height were measured, and BMI was calculated by weight (kg)/height^2^ (m). Physical activity was self-reported and estimated by two questions capturing physical exercise (six scores) and everyday physical activity (seven scores) [36]. A physical activity score was calculated by multiplying physical exercise with a factor of two (to consider higher intensity) and adding everyday physical activity resulting in an individual score of 3–19. Physical activity scores were divided into tertiles.

### 4.4. Data Acquisition

#### 4.4.1. Sampling, Sample Handling, and Preprocessing

Fasting venous blood was collected at one time point. Sample handling and preprocessing have been reported in detail elsewhere [31]. Briefly, blood was drawn into a 5 mL BD vacutainer glass tube, allowed to clot for 30 min, and centrifuged (2600× *g*, 10 min). Serum aliquots were placed at −20 °C within 1 h and stored at −80 °C within 2 h until analysis. Before ^1^H-NMR analysis, serum samples were thawed and mixed with phosphate buffer whereafter 180 µL of the sample mix was transferred to 3.0-mm NMR tubes (Bruker BioSpin, Billerica, MA, USA, 96 sample racks for SampleJet) using a SamplePro liquid handling robot (Bruker BioSpin). Samples were kept at 6 °C until analysis.

#### 4.4.2. NMR Spectroscopy

^1^H-NMR analysis has been described in detail previously [31]. In short, spectra were recorded at 800 MHz with a Bruker Advance III HD spectrometer with a 3-mm TCI cryoprobe. NMR data were recoded using the Bruker pulse sequence “zgespe”. A total of 128 scans were collected into 64 k data points. Data processing was performed with TopSpin 3.2p16 (Bruker BioSpin) and MatLab (MathWorks Inc., Natick, MA, USA), using TSP-d4 for referencing. In total 237 peaks were manually aligned and integrated peak-by-peak, and these variables represent ∼70 metabolites. A variable could also reflect more than one metabolite. Only variables of interest were identified.

For annotation Chenomx NMR suite 8.31 (Chenomx Inc., Edmonton, AB, Canada), the Human Metabolome Database [37] and an in-house implementation of the statistical total correlation spectroscopy (STOCSY) routine [38] were used.

### 4.5. Statistics

Subject characteristics and reported dietary intake are presented as median (min-max), mean (SD), or proportions (*n*, %). Partial correlation (Spearman’s rank correlation) was used to determine associations between foods and metabolites controlling for age (y, continuous), sex, physical activity score (categorical), BMI (kg/m^2^, continuous), and reported energy intake (kcal/d, continuous). Correlations were considered statistically significant if *p* values were <0.01. We further set absolute values of the correlation coefficients (rho) >0.2 to be considered relevant. Furthermore, to evaluate the predictive accuracy of dietary biomarkers to discriminate consumers from non-consumers, the area under the curve (AUC) was calculated from the receiver operating characteristics (ROC) curve. AUC <0.7 was deemed as low, 0.7 to <0.8 as moderate and ≥0.8 as high predictive accuracy. Because selected variables had to meet these three statistical requirements, no other correction for multiple hypothesis tests were done.

To assess if combined biomarkers would increase predictive models, a stepwise forward logistic regression was applied. All metabolites with an absolute correlation coefficient > 0.2 and *p* < 0.01 were included in the regression model. If two or more metabolites were included in the final model of stepwise forward logistic regression, these were further evaluated in combination using predictive probabilities. Diet–metabolite associations for combined biomarkers were evaluated using Spearman’s partial correlation. Further, AUC was calculated for the combined model and compared with the AUC for each single metabolite and ROC presented.

The computer software package SPSS for Windows, version 28 (IBM, New York, NY, USA) was used for statistical analyses.

## 5. Conclusions

Our results show that few serum metabolites are unique for a certain food item, but they can possibly be used in combinations to predict intake of some foods or food groups ingested in a habitual diet. The overall protein intake seems to be crucial for many of the metabolites found by ^1^H-NMR -analysis to associate with different foods. However, since many metabolites from animal products are both provided by the diet and are endogenously produced, it is important to adjust for factors such as lean body mass, which could contribute to the metabolite concentration in serum. Combinations of metabolites associated to food intake identified here might be of interest to evaluate further in dose–response intervention studies as potential combinatorial biomarkers. Finally, as the data analysis was performed without correction for multiple testing, we view the results presented here as an exploratory report on a potential method to combine metabolites from ^1^H-NMR -analysis to predict the intake of foods from serum samples.

## Figures and Tables

**Table 1:** Serum metabolites associated with reported food intake among healthy Swedish subjects (*n* = 119).

Food/Food Group	Metabolite Identification	Rho ^1^	*p* ^2^	AUC ^3^
SOY				
Soy beverage	Glycine	0.319	<0.001	0.710
Soy beans	(Asparagine) ^4^	0.252	0.007	0.891
	Valine	0.264	0.005	0.904
Soy products	Glycine	0.442	<0.001	0.776
Total soy	Glycine	0.435	<0.001	0.761
FRUIT AND VEGETABLES				
Cruciferous vegetables	Glutamine + unidentified	0.499	<0.001	0.721
Citrus fruits and marmalade	U ^5^ 21	0.503	<0.001	0.754
Total citrus fruits and juices	U21	0.628	<0.001	0.743
NUTS AND ALMONDS				
Nuts and almonds	U6	0.351	<0.001	0.714
	U2	0.397	<0.001	0.745
RED MEAT AND POULTRY				
Red meat	(3-methylhistidine)	0.265	0.004	0.701
	Creatine	0.511	<0.001	0.800
	U6	0.315	<0.001	0.739
	Valine + unidentified	0.345	<0.001	0.753
	Phosphocholine + acetylcholine + phosphoethanolamine + lipids/ffa	0.274	0.003	0.704
	Creatinine + ornithine	0.286	0.002	0.721
	Creatine + lysine	0.535	<0.001	0.824
	Arginine + lysine	0.304	0.001	0.727
	3-hydroxyisobutyrate	0.374	<0.001	0.768
	Valine	0.391	<0.001	0.769
	(2-aminobutyrate)	0.277	0.003	0.718
	Leucine	0.333	<0.001	0.742
	Leucine + isoleucine	0.269	0.004	0.706
	Isoleucine	0.249	0.008	0.704
Meat products/Processed meat	Creatinine	0.303	0.001	0.713
	Creatine	0.434	<0.001	0.778
	Creatinine + ornithine	0.268	0.004	0.706
	Creatine + lysine	0.422	<0.001	0.777
	3-hydroxyisobutyrate	0.331	<0.001	0.749
	Leucine	0.283	0.002	0.701
Poultry	Creatine	0.518	<0.001	0.870
	U5	0.335	<0.001	0.743
	Valine + unidentified	0.343	<0.001	0.751
	Creatine + lysine	0.524	<0.001	0.886
	3-hydroxyisobutyrate	0.384	<0.001	0.819
	Valine	0.380	<0.001	0.764
	Leucine	0.306	<0.001	0.721
	Leucine + isoleucine	0.303	0.001	0.700
Total meat	Creatinine	0.364	<0.001	0.724
	Creatine	0.553	<0.001	0.805
	Valine + unidentified	0.344	<0.001	0.730
	Valine + unidentified	0.382	<0.001	0.750
	Creatinine + ornithine	0.322	<0.001	0.721
	Creatine + lysine	0.551	<0.001	0.814
	Arginine + lysine	0.328	<0.001	0.730
	3-hydroxyisobutyrate	0.396	<0.001	0.765
	Valine	0.435	<0.001	0.765
	(2-aminobutyrate)	0.289	0.002	0.728
	Leucine	0.382	<0.001	0.753
	Isoleucine	0.290	0.002	0.720
SEAFOOD				
Fatty fish	Creatine	0.314	<0.001	0.747
	Creatine + lysine	0.283	0.002	0.733
Lean fish	Creatine + lysine	0.274	0.003	0.716
Shellfish	Creatine	0.245	0.009	0.706
Total fish and shellfish	Creatine	0.414	<0.001	0.748
	Creatine + lysine	0.443	<0.001	0.770
	3-hydroxyisobutyrate	0.302	0.001	0.709
EGGS				
Eggs	Creatine + lysine	0.334	<0.001	0.700
	3-hydroxyisobutyrate	0.370	<0.001	0.728
	Valine	0.418	<0.001	0.723
	(2-aminobutyrate)	0.353	<0.001	0.719
	Leucine	0.436	<0.001	0.735
	Isoleucine	0.364	<0.001	0.712
DAIRY				
Milk	Creatine	0.420	<0.001	0.730
	Creatine + lysine	0.480	<0.001	0.780
	Arginine + lysine	0.391	<0.001	0.741
	3-hydroxyisobutyrate	0.289	0.002	0.701
Fermented dairy products	Creatine	0.371	<0.001	0.726
	Valine + unidentified	0.353	<0.001	0.705
	Creatine + lysine	0.452	<0.001	0.777
	Arginine + lysine	0.287	0.002	0.713
	Valine	0.407	<0.001	0.714
	(2-aminobutyrate)	0.303	0.001	0.715
Cheese	Creatine + lysine	0.328	<0.001	0.743
	(2-aminobutyrate)	0.309	<0.001	0.708
Total dairy	Creatine	0.458	<0.001	0.749
	Valine + unidentified	0.333	<0.001	0.700
	Valine + unidentified	0.394	<0.001	0.723
	Creatine + lysine	0.524	<0.001	0.804
	Arginine + lysine	0.355	<0.001	0.743
	3-hydroxyisobutyrate	0.357	<0.001	0.736
	Valine	0.442	<0.001	0.747
	(2-aminobutyrate)	0.330	<0.001	0.732
	Leucine	0.416	<0.001	0.724
	Isoleucine	0.384	<0.001	0.714
ALCOHOLIC BEVERAGES				
Beer	Glucose + lysine + unidentified	0.258	0.005	0.718
	Isoleucine + unidentified	0.249	0.007	0.735
	Glucose	0.250	0.007	0.707
	Lipids/ffa + (methylguanidine)	0.378	<0.001	0.799
	Lipids/ffa	0.244	0.009	0.758
	Proline	0.253	0.007	0.709
	Proline + unidentified	0.272	0.003	0.752
White wine	(Xylose)	0.244	0.009	0.702
Spirits	Lactate + proline + 3-hydroxybutyrate	0.260	0.005	0.844
	Citrate	0.251	0.007	0.782

^1^ Spearman’s rank correlation coefficient; ^2^ Associations were calculated using Spearman’s partial correlation adjusted for age, sex, BMI, physical activity, and energy intake; ^3^ Area under the curve; ^4^ Uncertain metabolite identification in brackets; ^5^ Unidentified metabolite.

**Table 2:** Combined metabolite models to predict reported food intake among healthy Swedish subjects (*n* = 119).

Food/Food Group	Metabolite Identification	Rho ^1^	*p* ^2^	AUC ^3^
Soy beans	(Asparagine) ^4^	0.252	0.007	0.891
	Valine	0.264	0.005	0.904
	Combined metabolite model	0.301	0.001	0.967
Citrus fruits and marmelade	U ^5^ 22	0.248	0.008	0.637
	U21	0.503	<0.001	0.754
	Combined metabolite model	0.515	<0.001	0.780
Nuts and almonds	U4	0.299	0.001	0.696
	U2	0.397	<0.001	0.745
	Combined metabolite model	0.442	<0.001	0.749
Green/herbal tea	Asparagine	0.255	0.006	0.641
	Glycine	0.242	0.009	0.643
	Combined metabolite model	0.306	<0.001	0.686
Red meat	(3-methylhistidine)	0.265	0.004	0.701
	Creatine + lysine	0.535	<0.001	0.824
	Leucine	0.333	<0.001	0.742
	Combined metabolite model	0.631	<0.001	0.924
Meat products/Processed meat	Creatinine	0.303	0.001	0.713
	Creatine + lysine	0.422	<0.001	0.777
	Combined metabolite model	0.482	<0.001	0.849
Poultry	Creatine + lysine	0.524	<0.001	0.886
	Valine	0.380	<0.001	0.764
	Combined metabolite model	0.577	<0.001	0.939
Meat total	Creatinine	0.364	<0.001	0.724
	Creatine + lysine	0.551	<0.001	0.814
	Valine	0.435	<0.001	0.765
	Combined metabolite model	0.681	<0.001	0.932
Fish and shellfish total	Creatine + lysine	0.443	<0.001	0.770
	Valine	0.250	0.007	0.659
	Combined metabolite model	0.416	<0.001	0.772
Eggs	Valine + unidentified	0.316	<0.001	0.680
	Creatine + lysine	0.334	<0.001	0.700
	Valine	0.418	<0.001	0.723
	(2-aminobutyrate)	0.353	<0.001	0.719
	Combined metabolite model	0.591	<0.001	0.838
Milk	Creatine + lysine	0.480	<0.001	0.780
	Arginine + lysine	0.391	<0.001	0.741
	Combined metabolite model	0.532	<0.001	0.818
Fermented dairy	Creatine + lysine	0.452	<0.001	0.777
	Valine	0.407	<0.001	0.714
	Leucine + isoleucine	0.241	<0.001	0.611
	Combined metabolite model	0.577	<0.001	0.847
Cheese	Creatine + lysine	0.328	<0.001	0.743
	(2-aminobutyrate)	0.309	<0.001	0.708
	Combined metabolite model	0.372	<0.001	0.762
Dairy total	Creatine + lysine	0.524	<0.001	0.804
	Valine	0.442	<0.001	0.747
	(2-aminobutyrate)	0.330	<0.001	0.732
	Leucine + isoleucine	0.266	0.004	0.626
	Combined metabolite model	0.645	<0.001	0.889
Beer	Isoleucine + unidentified	0.249	0.007	0.735
	Lipids/ffa+ (methylguanidine)	0.378	<0.001	0.799
	Lipids/ffa	0.244	0.009	0.758
	Proline	0.253	0.007	0.709
	Combined metabolite model	0.365	<0.001	0.835

^1^ Spearman’s rank correlation coefficient; ^2^ Associations were calculated using Spearman’s partial correlation adjusted for age, sex, BMI, physical activity, and energy intake; ^3^ Area under the curve; ^4^ Uncertain metabolite identification in brackets; ^5^ Unidentified metabolite.

## Data Availability

The data presented in this study are available on reasonable request and should be made to Helen M. Lindqvist, helen.lindqvist@gu.se. The data are not publicly available due to Swedish law.

## Supplementary Materials

The following supporting information can be downloaded at: https://www.mdpi.com/article/10.3390/metabo12100908/s1, Table S1: Serum metabolites associated with reported food intake; Table S2: Reported consumption of 24 foods/food groups.
